# Role of antenatal care in reducing the risk of postpartum acute kidney injury

**DOI:** 10.12669/pjms.40.3.7127

**Published:** 2024

**Authors:** Aurangzeb Afzal, Sidra Saleem, Roshina Anjum, Haleema Tayyab

**Affiliations:** 1 Aurangzeb Afzal, MBBS, FCPS. Lahore General Hospital, Lahore, Pakistan; 2 Sidra Saleem, MBBS, FCPS, MRCP UK. Lahore General Hospital, Lahore, Pakistan; 3 Roshina Anjum, MBBS, FCPS. Lahore General Hospital, Lahore, Pakistan; 4 Haleema Tayyab, MBBS. Lahore General Hospital, Lahore, Pakistan

**Keywords:** Antenatal care, Postpartum, Acute kidney Injury

## Abstract

**Objective:**

Antenatal visits play a very important role to diagnose and manage pregnancy related health issues. This study was an attempt to identify the reasons that increase the risk of postpartum acute kidney Injury with special focus on antenatal care.

**Methods:**

We analyzed 110 patients in Nephrology and Gynaecology wards in Lahore General Hospital. Out of these 40 had Postpartum Acute Kidney Injury and 70 patients did not have it. Questionnaire regarding aspects of antenatal care (demographics, timing and number of antenatal visits) was filled by the patient or immediate family members.

**Results:**

Mean age of the 110 patients was 26.45 years. Mean Duration of pregnancy in the control group was 36.12 weeks and in cases it was 31.62 weeks. Out of 110 patients, 36(32.72%) patients did not have any antenatal visit while 62(56.3%) patients had more than five visits. Out of the 40 Postpartum Acute Kidney Injury patients, 23(57.5%) patients did not get any antenatal care. Out of 70 patients without Postpartum Acute Kidney Injury, 13 did not have any antenatal care. There were 19 patients who did not have booked visits because of financial Issues, followed by lack of awareness in 12 patients, distance issues for three patients and lack of family support for two patients.

**Conclusion:**

Patients who did not have antenatal care were at an increased risk of developing PPAKI. Financial issues and lack of awareness were the most common risk factors for compromised antenatal care.

## INTRODUCThION

Pregnancy related complications are an important cause of morbidity and mortality in pregnant females and babies.^1^ In developed countries risk of complications has progressively decreased in the last half century due to significant improvement in obstetric care. However, the picture is different in developing countries.^2^ In fact it differs in different parts of one country as well due to multiple factors such as availability of health facilities and awareness related to antenatal care etc.

In a developing country like Pakistan where a significant number of population is living in rural areas, pregnant women struggle for adequate health care. Pregnancy related Acute Kidney Injury still contributes significantly to maternal morbidity and mortality. Antenatal visits play a very important role to diagnose and manage pregnancy related health issues and so positively affect the maternal health and fetal outcome.^3^ Every year five million women become pregnant in Pakistan out of which fifteen percent face obstetrical and medical complications. Almost thirty thousand die due to pregnancy related complications every year.^4^ Postpartum AKI is one of the most important pregnancy related complication. It comprises 60% of pregnancy related AKI and has 19% maternal mortality rate.^5^ Despite this strikingly high number of maternal mortality only 37% pregnant women in Pakistan have three to four antenatal visits.^6^ Many studies have showed that improving antenatal care improves the outcome of pregnancy.^7^ This study was an attempt to identify the reasons that increase the risk of postpartum AKI with special focus on antenatal care so that we can alleviate all possible factors to minimize the risk of post-partum AKI. The objective of our study is to highlight the need for better quality of maternal care and fetal monitoring to decrease the morbidity and mortality related to PPAKI. Additionally, we aimed to identify the reasons and patient’s characteristics associated with their respective decision of not seeking medical help during pregnancy.

## METHODS

We analyzed 110 patients in Nephrology and Gynaecology wards in Lahore General Hospital. Out of these 40 had PPAKI and 70 patients did not have PPAKI. Informed consent was taken and a questionnaire regarding antenatal care was filled by the patient or immediate family members. The questionnaire obtained information about the aspects of care provided during pregnancy, the timing of the first ANC check-up, the number of ANC visits made during the last pregnancy and women’s socio-economic and demographic characteristics.

### Ethical Approval:

The study was approved by the hospital Ethics Committee on September 1st, 2019. (Ref. No. 00/04/21.

PPAKI was defined as AKI diagnosed from the time of childbirth to six weeks post-delivery. AKI was diagnosed and scored according to the KDIGO (Kidney Disease Improving Global Outcomes) classification based upon the rise in serum creatinine and /or the decrease in urine output during hospital Stay. Data was analyzed using SPSS version-23. Chi-square test was used to find the factors associated with the outcome. Statistical significance was tested at p < 0.05.

## RESULTS

Mean age of the 110 patients was 26.45 years with a range of 17-40 years and standard deviation of 4.922. The average duration of pregnancy was 34.76 ± 4.073 weeks. Mean Duration of pregnancy in the control group was 36.12 weeks and in cases it was 31.62 weeks. Out of 110 patients, 36(32.72%) patients did not have any antenatal visit. Sixty two (56.3%) patients had more than five visits. Out of the 40 PPAKI patients, 23(57.5%) patients did not get any antenatal care. Out of 70 patients without PPAKI 13 did not have any antenatal care as shown in Table-I.

**Table-I:** Comparison of Antenatal Visits in Patients with PPAKI and without PPAKI.

Antenatal visits	All patients (110)	Cases (40)	Control (70)
0	36(32.72 %)	23(57.5%)	13(18.5%)
1-2	10(9.09 %)	2(5%)	8(11.4%)
3-5	2(1.8%)	(2.5%)	1(1.4%)
>5	62(56.36%)	14(35%)	48(68.5%)

Independent t-test was applied to make comparison of antenatal care between controls and cases as shown in Table-II. We have applied levene’s test in first portion which has *p-value* less than 0.05 which shows that variances are not equal. And in next portion p-value is less than 0.05 which shows there is significant difference between controls and cases. It means those pregnant women who do not get antenatal care for some reason have more risk of PPAKI.

**Table-II:** Comparison of antenatal visits between Cases and Control-Independent T-Test.

Levene’s Test		P-Value
F-test	P-Value	0 .000
21.674	.000	

There were 19 patients in total who did not have booked visits because of financial Issues, followed by lack of awareness in 12 patients. Three patients did not go to the hospital/clinic for antenatal care because it was far away from their house and two patients did not have any family support to visit hospital/clinic for antenatal care as shown in Table-III. Significant association was found between above mentioned factors and no antenatal care with a p-value of less than 0.5.

**Table-III:** Reason for no booked visit to the doctor.

Reason for no booked visit	All patients (36)	Cases (23)	Control (13)
Lack of awareness	12(33.3%)	6(26%)	6(46.1%)
Financial Issues	19(52.7%)	16(69.5%)	3(23%)
Far away from hospital	3(8.3%)	0	(23%)
No family support	2(5.5%)	1(4.3%)	1(7.6%)

Among the 74 patients with booked visits, only 56 got their tests done and 66 patients’ husbands had an income between PKR/- 10,000-20,000. Twenty four patients’ husbands had an income less than 10,000 PKR/- as shown in Table-IV.

**Table-IV:** Reasons lack of tests and antenatal care.

		All Patients	Case (40)	Control (70)
Patients with booked visits		74	17(42.5%)	57(81.4%)
Tests advised		70	16(40%)	54
Tests done		56	9(22.5%)	47(67.1%)
Husband income	<10,000	24(21.8%)	15(37.5%)	9(12.8%)
	10,000-20,000	66(60%)	22(55%)	44(62.8%)
	20,000-50,000	18(16.3%)	3(7.5%)	15(21.4%)
	>50,000	2(1.8%)	0	2(2.8%)
Husband cooperative		108		

Out of 110 patients, 47(42.7%) patients had SVD, 61(55.4%) had LSCS.21 (52.5%) patients with PPAKI had SVD whereas 18(45%) had LSCS. About 26(37.1%) patients in the control group had SVD, whereas 43(61.4%) had LSCS. One patient in each group had Dai handling. No significant association was found between place and mode of delivery and PPAKI.

Among the 40 PPAKI patients, only 8(20%) had alive babies and 32(80%) delivered babies with Intrauterine death (IUD). Those who did not have PPAKI, 63(90%) delivered alive healthy babies and 7(10%) delivered IUD babies. Chi square test was applied and it showed positive association between peripartum AKI and perinatal mortality with a p-value of less than 0.05.

## DISCUSSION

Maternal morbidity and mortality are serious health issues in developing countries like Pakistan. Good antenatal care can significantly reduce the maternal and perinatal mortality as many of the pregnancy related complications are either preventable or treatable if diagnosed early. To our knowledge this is perhaps one of very few studies done in last decade, in this part of the world, on factors responsible for inadequate antenatal care and role of antenatal care in prevention of postpartum AKI.

Out of 110 patients, 36(32.72%) patients did not have any antenatal care while 62(56.3%) patients had more than five visits. Out of the 40 PPAKI patients, 23(57.5%) patients did not get any antenatal care suggesting that number of antenatal visits is indirectly proportional to incidence of PPAKI. In another study done in pregnancy related AKI patients it was found that only 20% patient had proper antenatal care by a gynecologist.^8^ A study done in India also concluded that out of 57 patients with pregnancy related AKI less than half received antenatal care.^9^ Mean age of the patients was 26.45 years which is comparable with a study done by Bokhari et al.^8^

In our study we found that among 40 cases of PPAKI 80% pregnancies ended up with intrauterine death (IUD), in contrast to control group where IUDs were only 10%. Perinatal mortality was 3.4 folds higher in cases with pregnancy related AKI as compared to those without PAKI as stated in one study.^10^ In many studies it has been proven that pregnancy related AKI affects the outcome of pregnancy significantly. It can end up with pre term birth, IUD or neonatal death.^1^,^11^,^12^

Mean Duration of pregnancy in the control group was 36.12 weeks. In cases it was 31.62 weeks that is a pre-term birth. Pre term birth is defined as birth between 20-37 weeks of gestation.^13^ Last trimester of pregnancy is very vital in development of fetal kidney as more than half nephron develops during this phase. Since Pre term babies are born with smaller number of nephrons they are at increased risk of development of CKD in their later life. Additionally, they have more chances to develop diabetes, cardiovascular disease and metabolic syndrome as well.^14^-^16^ So it is clear that diagnosis and management of obstetrical complications not only reduce the perinatal mortality but also co morbidities in later life as well making it even more important.

In this study we tried to find out the reasons for compromised antenatal care. For that we interviewed our patients according to a pre designed questionnaire and it was seen that 19(52.7%) out of 36 patients who had not booked visits were having financial issues. Effect of socioeconomic status on antenatal care utilization has been studied well in past and results showed a direct relation between good financial status and utilization of antenatal care.^17^,^18^ Our study results also endorsed the similar idea.

Knowledge and awareness about benefits of antenatal care is the key to persuade mothers to attend antenatal clinics. In Pakistan where most of the population is rural with limited access to health care, lack of awareness is an important factor. Many people believe that as pregnancy is a natural process so it does not need medical assistance. In our study lack of awareness was the second most common reason of inadequate antenatal care highlighting the need of education and public awareness related to importance of antenatal care. Role of women’s knowledge about antenatal health and danger signs of pregnancy in improving the antenatal care has been proven in many other studies as well.^19^,^20^

In our study an important reason of missed antenatal visits was distance from health care facility. In many studies it has been proven that distance from healthcare facility is an important barrier causing significant reduction in number of antenatal visits.^21^ In Pakistan only 33% of rural population have antenatal healthcare facility within five kilometer radius according to a survey done in past.^22^ In rural areas where usually pregnant females needs a family member to accompany them and have to bear transport charges so even this distance becomes a hindrance in seeking medical help.

Lack of support from family or spouse significantly affects the attendance in antenatal clinic.^23^ It has been proven in our study as well in addition to many studies supporting the same theory in the past.^24^,^25^ This study is an eye opener for us to do some necessary arrangements for antenatal services is Pakistan. It can serve as a reference for future studies in other centers in our part of the world so that we can work at an individual and government level to educate our masses about danger signs & role of antenatal care and to enhance our health care facilities especially for women of child bearing age who are the back bone of our society.

### Limitations:

This is a single centered study and includes a set of patients from a government hospital. More studies are needed from other centers with patients from different socioeconomic background to compare the data.

## CONCLUSION

Patients who did not have antenatal care were at an increased risk of developing PPAKI. Financial issues and lack of awareness were the most common risk factors for compromised antenatal care.

### Author’s contribution:

**AA**: Contribution in conception, design of study, acquisition and interpretation of data, drafting of manuscript, research coordination and management.

**SS**: Conception and design, drafting of manuscript, revising and editing the manuscript.

**RA**: Drafting, editing and revising the manuscript.

**HT**: Conception and design of study, drafting of manuscript, revising and editing the manuscript research coordination and management.

## References

[1] Mahesh E, Puri S, Varma V, Madhyastha PR, Bande S, Gurudev KC (2017). Pregnancy-related acute kidney injury:An analysis of 165 cases. Indian J Nephrol.

[2] Davison J (2005). Renal complications that may occur in pregnancy. Oxford Text B Clin Nephrol.

[3] Majrooh MA, Hasnain S, Akram J, Siddiqui A, Memon ZA (2014). Coverage and quality of antenatal care provided at primary health care facilities in the Punjab province of Pakistan. PLoS One.

[4] Khan YP, Bhutta SZ, Munim S, Bhutta ZA (2009). Maternal health and survival in Pakistan:issues and options. J Obstet Gynaecol canada.

[5] Eswarappa M, Madhyastha PR, Puri S, Varma V, Bhandari A, Chennabassappa G (2016). Postpartum acute kidney injury:a review of 99 cases. Ren Fail.

[6] Ashraf F, Thaver IH, Imtiaz F, Ayub A (2017). Quality assessment of focused antenatal care service delivery in tertiary care health facility. J Ayub Med Coll Abbottabad.

[7] Coria-Soto IL, Bobadilla JL, Notzon F (1996). The effectiveness of antenatal care in preventing intrauterine growth retardation and low birth weight due to preterm delivery. Oxford University Press.

[8] Bokhari SRA, Inayat F, Jabeen M, Sardar Z, Saeed S, Malik AM (2018). Characteristics and Outcome of Obstetric Acute Kidney Injury in Pakistan:A Single-center Prospective Observational Study. Cureus.

[9] Godara SM, Kute VB, Trivedi HL, Vanikar A V, Shah PR, Gumber MR (2014). Clinical profile and outcome of acute kidney injury related to pregnancy in developing countries:a single-center study from India. Saudi J Kidney Dis Transplant.

[10] Liu Y, Ma X, Zheng J, Liu X, Yan T (2017). Pregnancy outcomes in patients with acute kidney injury during pregnancy:a systematic review and meta-analysis. BMC Pregnancy Childbirth.

[11] Prakash J, Pant P, Prakash S, Sivasankar M, Vohra R, Doley PK (2016). Changing picture of acute kidney injury in pregnancy:Study of 259 cases over a period of 33 years. Indian J Nephrol.

[12] Hildebrand AM, Liu K, Shariff SZ, Ray JG, Sontrop JM, Clark WF (2015). Characteristics and outcomes of AKI treated with dialysis during pregnancy and the postpartum period. J Am Soc Nephrol.

[13] Robinson JN, Norwitz ER (2019). Preterm birth:Risk factors, interventions for risk reduction, and maternal prognosis. UpToDate, Waltham, MA Sott 26.

[14] Piccoli GB, Zakharova E, Attini R, Ibarra Hernandez M, Covella B, Alrukhaimi M Acute kidney injury in pregnancy:The need for higher awareness. A pragmatic review focused on what could be improved in the prevention and care of pregnancy-related AKI, in the year dedicated to women and kidney diseases. J Clin Med.

[15] Weight TLB, Group NNW, others (2017). The impact of kidney development on the life course:a consensus document for action. Nephron Clin Pract.

[16] Luyckx VA, Brenner BM (2015). Birth weight, malnutrition and kidney-associated outcomes a global concern. Nat Rev Nephrol.

[17] Efendi F, Chen C-M, Kurniati A, Berliana SM (2017). Determinants of utilization of antenatal care services among adolescent girls and young women in Indonesia. Women Health.

[18] Zhao Q, Huang ZJ, Yang S, Pan J, Smith B, Xu B (2012). The utilization of antenatal care among rural-to-urban migrant women in Shanghai:a hospital-based cross-sectional study. BMC Public Health.

[19] Onasoga OA, Afolayan JA, Oladimeij BD Factor's influencing utilization of antenatal care services among pregnant women in Ife Central LGA, Osun State Nigeria. Adv Appl Sci Res.

[20] Birmeta K, Dibaba Y, Woldeyohannes D Determinants of maternal health care utilization in Holeta town, central Ethiopia. BMC Health Serv Res.

[21] Yamashita T, Kunkel SR The association between heart disease mortality and geographic access to hospitals:county level comparisons in Ohio, USA. Soc Sci Med.

[22] Lin G, Allan DE, Penning MJ (2002). Examining distance effects on hospitalizations using GIS:a study of three health regions in British Columbia, Canada. Environ Plan A.

[23] Ali SA, Dero AA, Ali SA others. Factors affecting the utilization of antenatal care among pregnant women:a literature review. J Preg Neonatal Med.

[24] Gross K, Alba S, Glass TR, Schellenberg JA, Obrist B (2012). Timing of antenatal care for adolescent and adult pregnant women in south-eastern Tanzania. BMC Pregnancy Childbirth.

[25] Rosliza AM, Muhamad JJ Knowledge, attitude and practice on antenatal care among orang asli women in Jempol, Negeri Sembilan. Malaysian J Public Heal Med.

